# Assessment of the Impact of Superficial Contamination and Thermo-Oxidative Degradation on the Properties of Post-Consumer Recycled Polypropylene

**DOI:** 10.3390/ma16031198

**Published:** 2023-01-31

**Authors:** Laura Prior, Mónica S. A. Oliveira, Tatiana Zhiltsova

**Affiliations:** 1 Department of Mechanical Engineering, Centre for Mechanical Technology and Automation (TEMA), University of Aveiro, 3810-193 Aveiro, Portugal; 2 LASI—Intelligent Systems Associate Laboratory, 4800-058 Guimaraes, Portugal

**Keywords:** polymers, recycling, degradation, decontamination by washing, post-consumer, mechanical properties

## Abstract

Single-use plastics are a matter of convenience in everyday life, with the majority allocated to packaging production. However, it comes with a high environmental price as its mass recycling is challenging due to the heterogeneity of composition, contaminations of different kinds, and degradation caused by service and processing. This study aims to ascertain the impact of removing contaminants from post-consumer recycled polypropylene (rPP) on its degradation and properties by implementing a systematic approach for decontamination by washing. Four lots of recycled plastics with different degrees of contamination were evaluated via Fourier transform infrared, melt flow indexer, and differential scanning calorimetry and tested for tensile strength. Degradation of the rPP was manifested by the deterioration in ductility, resulting in 14.58% elongation at break (unwashed rPP) compared with 191.41% (virgin PP)) and a significant reduction in oxidation induction time. In the unwashed rPP sample, a wave intensity peak at 1730 cm^−1^, assigned to the saturated C = 0 stretch of the carbonyl functional group, was detected. This peak was gradually disappearing with an increase in the cleaning efficiency of rPP, highlighting the role of contaminants as degradation catalysts. The cold-washing method showed similar processing and mechanical performance improvement results compared with the other washing methods, while being more environmentally friendly and energy efficient.

## 1. Introduction

Polymers became an indispensable part of modern society, revolutionizing, simplifying, and improving the way of living and being. They combine low price and weight with high versatility of applications and processability, thus, becoming preferred materials for plastic food and non-food packaging, enabling better and safer conditioning of goods and, subsequently, reducing waste [1,2,3]. However, despite a steady increase in post-consumer plastic collection over recent years, only a third of this amount is effectively recycled, and the remainder is used either for energy recovery or disposed of in landfills [4]. Such a low rate of recycling may be explained by a quality issue inherent to post-consumer recycled plastics, which mainly originated from two sources: contamination either by immiscible polymer inclusions, by primary use, and by waste collection methods [5,6,7,8], and thermo-oxidative degradation occurring during synthesis, transformation, service life as a product, and reprocessing after recycling [9]. Thermo and photo-oxidative degradation is known to be a significant factor in the deterioration of recycled PP properties due to the mechanisms of molecular scission and the formation of oxidized moieties [10,11,12]. Determination of the degradation degree is essential to understanding recycled plastic products’ future performance. A decrease in the melting temperature of photodegraded PP was linked to progressively lower molecular weight, lower rates of nucleation in the crystalline structures, and a larger number of chemical irregularities in the form of carbonyl groups [13,14,15]. An increase in the degradation level of PP due to intensified molecular scission and, therefore, shorter molecular chains, which promote increased mobility at a molecular level, is reflected as a rise in the crystallization temperature [15,16]. Higher crystallization temperatures were associated with a greater number of crystalline particles with the γ form developed during the crystallization of molecules with low molecular weight due to degradation through molecular scission [17].

Alteration of crystallinity degree is another degradation signature of widely researched polymers. The decrease in PP crystallinity, according to Ojeda et al. [12], was attributed to the increased concentration of chemical impurities (i.e., carbonyl and hyperoxide groups) due to antioxidant consumption in PP. However, Rabello and White [13] show that the crystallization degree of PP may vary either way, increasing after short-term exposure to photo-oxidative degradation and then decreasing after prolonged exposure. Short-term exposure to light and oxygen reduces the molecular mass and increases polarity, leading to greater polymer chain mobility and promoting reorganization into more orderly structures. However, with more prolonged exposure, the crystallinity degree becomes limited due to chemical irregularities like carbonyl and hydroperoxides due to continuous photooxidation. Similar results are reported by Wu et al. [14] and Elvira et al. [16] that associate the decrease of crystallinity with the increase of carbonyl groups that form during the oxidation of PP. It should be stressed that one of the most notorious effects of PP degradation is a deterioration of the mechanical properties. The oxidative degradation in semicrystalline plastics, in general, and in PP, in particular, initiates in the crystalline interphase and advances through the amorphous regions. Therefore, when these tie molecules linking polymer crystallites undergo scission, their bearing capacity to withstand the applied stress is significantly diminished, leading to rapid embrittlement even at low oxidation levels [18,19]. These observations were supported by various authors [10,12,20,21], who reported a decrease in the mechanical properties of recycled polyolefins, especially the abrupt reduction in the elongation at break.

Along with the assessment of recycled post-consumer plastics degradation state, significant efforts were dedicated to the investigation of the influence of contaminants removal by washing on their properties [7,10,22,23,24,25,26,27]. Most of these studies were focused on odor removal by different washing processes, which is a well-known problem of post-consumer plastic originating from food and nonfood packaging caused when organic compounds adhere to the surface and are embedded into the polymer matrix [22,23,24]. Inks, partially consumed additives, and possible degradation products were identified as origins of odor [22]. The applied washing methods have proven to be effective in partially removing odor contaminants, as demonstrated by Demets et al. [23], where a reduction of odor constituents in contaminated plastic films (rinsed and washed in a friction washer) was detected. However, after extrusion and pelletizing, these contaminants’ content increased due to their release during melting and thermal degradation, being, nevertheless, lower than in unwashed plastic waste. Strangl et al. [28] reached a similar conclusion for extruded and pelletized post-consumer mixed packaging polyolefins. The presence of dirt in copolymer PP requires lower activation energy for 10% thermogravimetric weight loss compared with uncontaminated plastic, confirming the accelerating effect of degradation due to contaminants [29]. This conclusion was corroborated in the recent study of Veroneze et al. for artificially contaminated PP. They have reported that the residual contaminants decrease the molar mass and exacerbate the degradation reaction [27]. However, in the studies mentioned above, the impact of recyclates’ purity on the mechanical properties of post-consumer recycled plastics was not investigated. Very few studies systematically tackle this important topic. Garofalo et al. [7] reported an improvement in the flexural modulus of post-consumer polyethylene (PE) contaminated with PP due to the removal of low molecular compounds, especially evident when hot-water washing with caustic soda was implemented.

Despite the considerable amount of research dedicated to the post-consumer degradation of PP, it is still essential to carry out structured research mainly focused on evaluating the influence of superficially adhered contaminants responsible for its post-consumer degradation. Therefore, this study aims to investigate the impact of the contamination of PP, recovered from post-consumer plastic packaging waste, on its degradation by assessing the rheological, thermal properties and mechanical performance. To this end, three different washing procedures were implemented, resulting in four batches of recycled plastics with different degrees of contamination. These four batches and the reference virgin PP were investigated with FTIR, MFI, and DSC and tested for tensile strength to identify the influence of contamination on post-consumer PP degradation. The present work’s main contributions were identifying the most efficient washing method for the contaminants’ removal and clarifying the potential contribution of the contaminants to the degradation of the reprocessed post-consumer PP.

## 2. Materials and Methods

### 2.1. Materials

The material used in this study was a recycled PP collected from several distributed recycling locations via the yellow bin system. First, the retrieved material, mainly food and nonfood packaging, was sorted according to the plastic recycling code, and only PP waste was retained. After the sorting stage, it was coarsely cut by hand to simplify the grinding step, which was accomplished using a plastic grinder from PLASMAQ MRU 18-20 (Plasmaq Cm, Lda, Barrosa, Leiria, Portugal). The plastic was shredded three times until the flakes reached a granulometry lower than 4 mm, achieved by sieving through the mesh strainer (mesh size 4 mm). Recycled PP (rPP) flakes were divided into four batches during the third material preparation step. One of the batches was not washed, designated further as (rPPu). In post-consumer plastic waste, the most frequently encountered superficially adhered contaminants are dirt and organic residues, removable by washing. It was accomplished by subjecting three other batches to different washing procedures: washed with cold water (rPPcw), washed with hot water (rPPhw), and hot-washed with cleaning agents (rPPhwca), i.e., caustic soda (NaOH) and surfactant Triton X-100. Washing was accomplished with the mechanical stirrer Yellow Line OST 20 digital (IKA^®^-Werke GmbH & Co. KG, Staufen, Germany) with an impeller speed of 1300 rpm agitated for 10 min. After washing and separation via sink float method, drying was accomplished in the Meemert Universal Oven UF30 (Memmert GmbH + CO.KG, Schwabach, Germany) at 60 °C for 24 h. 

**Figure 1:**
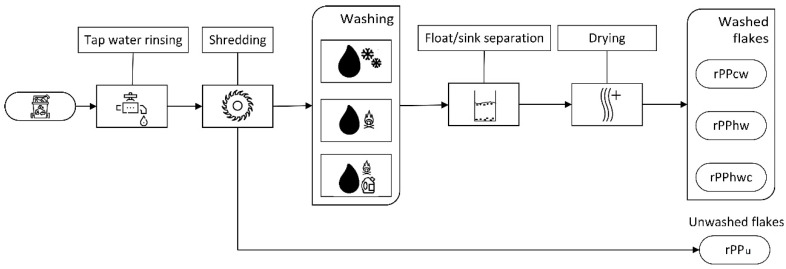
rPP flakes preparation procedure.

The washing and drying conditions for each recycled PP batch are reported in Table 1. A more detailed description of washing, sink float separation, and drying procedures may be consulted elsewhere [30]. The schematic of the transformation of the post-consumer PP waste into secondary raw material is demonstrated in Figure 1. Virgin PP Moplen HP500N (isotactic homopolymer) from LyondellBasell was used as a reference.

### 2.2. Methods

#### 2.2.1. Melt Flow Index

The measurements of the melt flow index were performed on the machine Göttfert MI-3 (GÖTTFERT Werkstoff-Prüfmaschinen GmbH, Buchen, Germany) according to the standard ASTM D1238-04 [31] with a temperature of 230 °C and a load of 2.16 kg. Ten samples were analyzed for each recycled PP batch and virgin PP.

#### 2.2.2. Differential Scanning Calorimetry

Melting (T_m_) and crystallization (T_c_) temperatures, and melting (ΔH_m_) and cold crystallization (ΔH_c_) enthalpies were obtained by differential scanning calorimetry (DSC) from samples with an approximate weight between 5 and 10 mg. DSC tests were conducted, based on the guidelines given by the standard ASTM D3418-03 [32], on DSC Discovery 250 (TA Instruments, New Castle, DE, USA), and data were treated with TRIOS software (proprietary software of TA Instruments). For each PP batch, three samples were analyzed. Each sample was exposed to two heating and cooling cycles, where the first one aimed to “erase” the thermal history of the material. Only the data from the second cycle was collected and analyzed. The samples were stabilized at 20 °C, then heated up to 200 °C, and cooled down to 20 °C at a heating and cooling rate of 10 °C/min. 

Each sample’s degree of crystallinity (χ) was calculated by equation 1 [33].

χ(%) = (∆H_m_/(∆H^0^_m_) × 100 (1)

where ΔH_m_ (J/g) is the melting enthalpy of the polymer under analysis, and the melting enthalpy of 100% crystalline PP (∆H^0^_m_) is 207 J/g [33].

Oxidation induction time was obtained according to the detailed procedure in ASTM D3895-14 [34]. PP samples were films covering the bottom of the aluminium crucible without a lid, with a mass between 5 and 10 mg. They were heated under a nitrogen atmosphere from ambient temperature up to 200 °C at a heating rate of 20 °C/min and maintained isothermally for 5 min. Next, N_2_ atmosphere was changed to an oxygen atmosphere and maintained until the exothermic peak was registered. OIT was determined as an intercept between the baseline of the oxidative reactive exotherm and its steepest linear slope. Three tests were performed for each PP batch.

#### 2.2.3. Infrared Spectroscopy (FTIR)

The FTIR spectra in transmittance mode were acquired within a wavenumber range from 4000 to 350 cm^−1^, using a resolution of 4 cm^−1^. A total of 256 spectra were acquired and averaged for a sample (i.e., individual injection molded specimen) tested for each PP batch. The ATR ZnSe crystal was cleaned after each scan with ethanol, and a new background calibration was performed. The measurements were carried out using a Bruker Tensor 27 FT-IR Spectrometer (Bruker Optik GmbH, Billerica, MA, USA).

#### 2.2.4. Mechanical Properties

For the tensile testing, ISO 527-2 [35] type 5 A specimens were obtained by injection molding with the mini injection machine HAAKE MiniJet II (Thermo Fisher Scientific, Waltham, MA, USA). The injection molding processing conditions of the virgin and recycled PP specimens are shown in Table 2.

The tensile tests were conducted on the machine Shimadzu AGS-X-10kN (Shimadzu Scientific Instruments (SSI), Columbia, MD, USA) following the standard ISO 527-1 [36]. These tests were executed at ambient temperature in two steps. First, the specimens were pulled with a tensile rate of 1 mm/min to obtain values for calculating the Young modulus. In the second stage, a tensile rate of 50 mm/min was applied and maintained until the specimens ruptured. The data from this second test was used to determine the yield stress (σ_y_) and strain (ε_y_), and tensile strength (σ_u_) and strain at break (ε_b_). It should be noted that the latter is especially relevant for the polymer degradation assessment because of this property’s extraordinary sensitivity to any structural change [37]. For each PP batch, five specimens were tested.

## 3. Results and Discussion

### 3.1. Melt Flow Index

**Figure 2:**
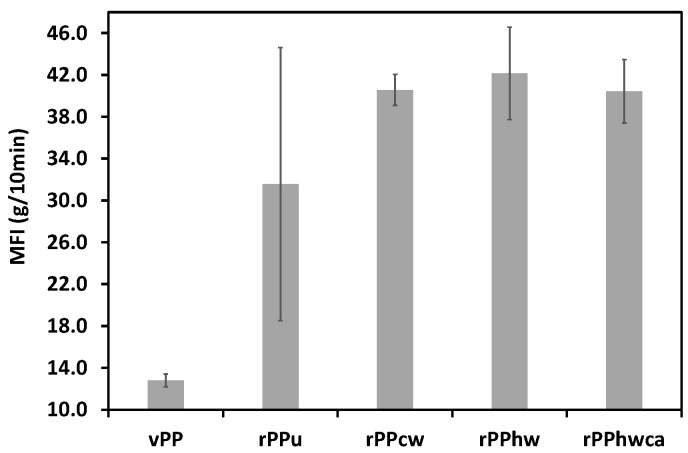
Melt flow index of virgin and recycled PP.

The melt flow index measurements for virgin and recycled PP with different levels of contamination are shown in Figure 2. The virgin PP has a melt flow index of 12.82 g/10 min, differing slightly from the reference (12 g/10 min) stated in the manufacturer’s datasheet. All recycled PP samples show higher MFI than the reference material (vPP). It should be mentioned that the recycled PP samples comprise a wide variety of PP grades for packaging applications of unknown origin, and their direct comparison in terms of MFI with virgin PP is not viable. MFI does not seem to be an adequate technique to ascertain the possibility of polymer degradation in this case. However, some conclusions can be drawn regarding the processability of recycled PP batches as their MFI varies from about 31.56 to 42.14 g/10 min, representative of medium and low viscosity grades, typical grades for food packaging, and, therefore, suitable for reprocessing by injection molding. The contaminated rPPu has a lower MFI of 31.6 g/10 min with a standard deviation of 13.04, i.e., higher melt flow index dispersity, which may generate difficulties during processing. It is worth mentioning that, according to the appearance of the wash water (dark grey) and to the presence of the sediments (dirt, paper particles, heavier plastic flakes) after float sink separation, superficially adhered contaminants present in post-consumer PP are mainly particulate contaminants. Two factors may explain the high standard deviation and lower MFI of unwashed samples: the uneven concentration of the particulate matter contaminants on the surface of the flakes and the fact that the particulate matter contaminants have a similar effect to that of a filler. After removing contaminants by washing, rPPcw, rPPhw, and rPPhwca samples had higher MFI and significantly lower dispersity of measurements than the rPPu. The mean MFI values of the three applied washing methods are quite similar, varying between 40.4 and 42.1 g/10 min, which is representative of reground of injection molding and blow molding packaging. 

It should be stressed that rPPcw, rPPhw, and rPPhwca have larger MFI dispersity than virgin PP, which is also expected and may be explained by the heterogeneity of recycled PP composition from sample to sample, leading to variations in the respective melt flow indexes. The material batch with the less standard deviation of 1.49 was rPPcw, probably indicating that the cold-water washing method, consuming less energy and with low environmental impact, is the most efficient for recycled PP rheological stability.

### 3.2. Differential Scanning Calorimetry 

#### 3.2.1. Thermal Transitions and Degree of Crystallinity

The relevant data concerning melting peak temperature T_m_, melting enthalpy ∆H_m_, crystallization peak temperature T_c_, and degree of crystallinity, obtained from DSC analysis, are compiled in Table 3.

**Figure 3:**
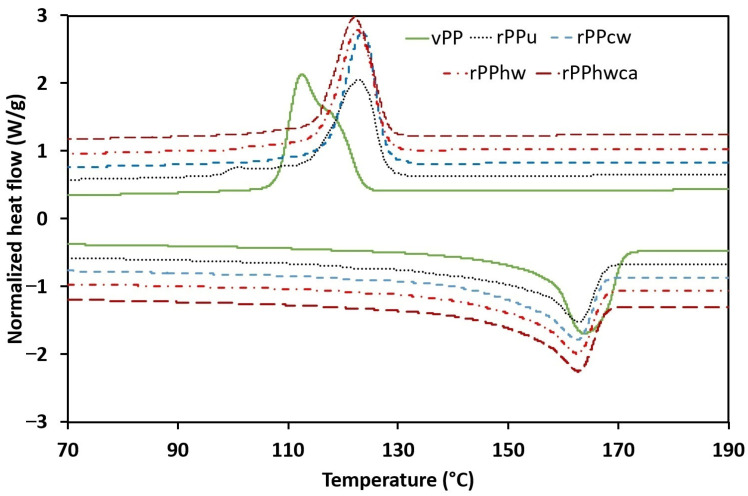
DSC curves for virgin and recycled PP (Upper curves from the second cooling scan and lower curves from the second heating scan have been arbitrarily shifted to improve comparability. Exothermal direction is up).

There is a slight decrease in the melting temperature in all recycled PP samples. The melting temperature between the different batches of recycled PP fluctuates within a range of 1.2 °C. Therefore, it is impossible to draw any definite conclusion about the impact of washing on the melting temperature. A considerable increase in the crystallization temperature was registered in all recycled PP samples compared to virgin PP (Figure 3). PP is a polymorphic material and can crystallize in different crystalline forms, the most common and stable being the α form, but the γ form is also likely to be found in PP and can even coexist with the α [38,39]. Therefore, this increase in crystallization temperature may be attributed to the degradation of recycled PP, which provokes molecular scission, resulting in shorter chains with lower molecular weight and, therefore, crystallization in γ form likely to occur at higher temperatures, as advocated by De Rosa et al. [17]. The difference in the peak crystallization temperature is barely perceptible between the different batches of washed and contaminated recycled PP. Therefore, no definite conclusions can be formulated regarding the influence of washing on the crystallization temperature.

The degree of crystallinity was reduced in all recycled PP samples, varying between 47 and 48.5% compared with vPP (52.5%), as shown in Table 3. These results are corroborated by the data reported by Ojeda et al. [12] and Rabello and White [13], who stated that degraded PP, after prolonged exposure to photo-oxidation, has lower crystallinity content. Regarding the influence of the removal of contaminants on the crystallinity, the highest value of 48.5% was obtained for rPPhwca, while the lowest crystallinity content was obtained for rPPhw. The latter results seem to be in contradiction with the data reported in the literature, as an increase in the crystallinity is to be expected with the removal of the contaminants. A decrease in crystallinity due to contaminants, acting as the hinders of chain packaging, has been reported by Fitaroni et al. [40] for PET samples. Veroneze et al. [27] related a decrease in the enthalpy of fusion, influenced by the diffusivity of the polymer chains to the presence of the contaminants. Nevertheless, only the values for rPPhw are out of the expected trend, while the crystallinity content of the other recycled PP batches slightly increases with decontamination. This out-of-trend value of rPPhw may be explained by the high standard deviation of the correspondent melting enthalpy compared with the other batches (Table 3), essentially due to a lack of homogeneity between the samples. 

#### 3.2.2. Oxidation Induction Time

**Figure 4:**
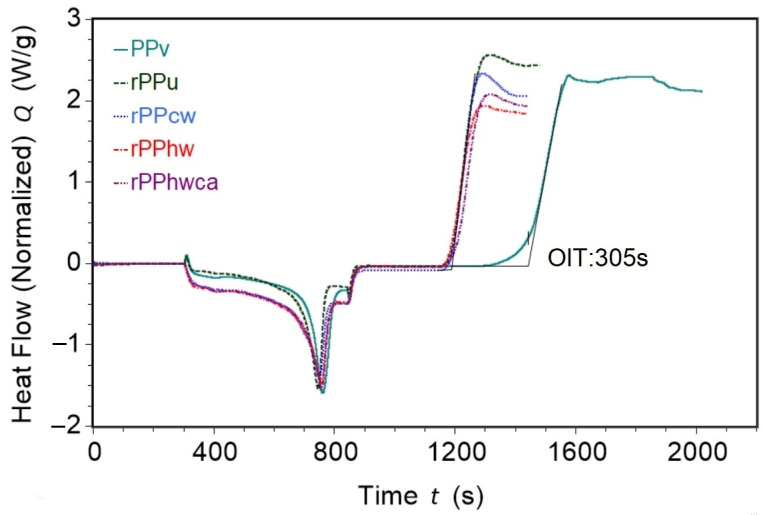
OIT curves of virgin-and recycled PP.

The oxidation induction time test allows accessing the level of stabilization of the material by determining the time of oxidative decomposition. As shown in Figure 4., all the recycled PP samples show considerably lower OIT when compared with vPP. The latter samples oxidize in pure oxygen in about 300 s. All the recycled PP samples show considerably lower OIT than vPP, indicating a significant reduction of the antioxidants’ content due to degradation processes induced primary by processing and service life of the constituent’s different PP grades. As a result, the polymer matrix of these materials is unprotected against oxidation, which leads to the deterioration of their properties and may compromise future applications. This result highlights the necessity of adding antioxidants to compensate, to some extent, for the deterioration of the post-consumer recycled plastics properties. As can be seen from Table 4, the oxidation induction times of all the recycled PP samples vary between 41.6 and 61.4 s. The latter time is the best performance shown by rPPhwca samples with the most thorough hot-washing treatment with cleaning agents, corroborating various researchers’ conclusions [27,29,41] about the accelerating effect of thermo-oxidative degradation due to the presence of contaminants. Besides the antioxidant’s destruction and influence of the particulate contaminants, which cannot be entirely removed by washing, the concentration, and composition of residual polymerization catalysts and metal additives may contribute to the formation of degradation products and, thus, decrease the thermo-oxidative stability of PP [42]. The increasing concentration of metals associated with polymerization catalysts in post-consumer PP was demonstrated by Curtzwiler et al. [43].

It also should be noted that high variability of OIT for virgin PP was detected, which could originate from the storage conditions, leading in turn to its uneven oxidation, reflected as the measurements’ dispersity.

### 3.3. FTIR Spectroscopy Analysis 

**Figure 5:**
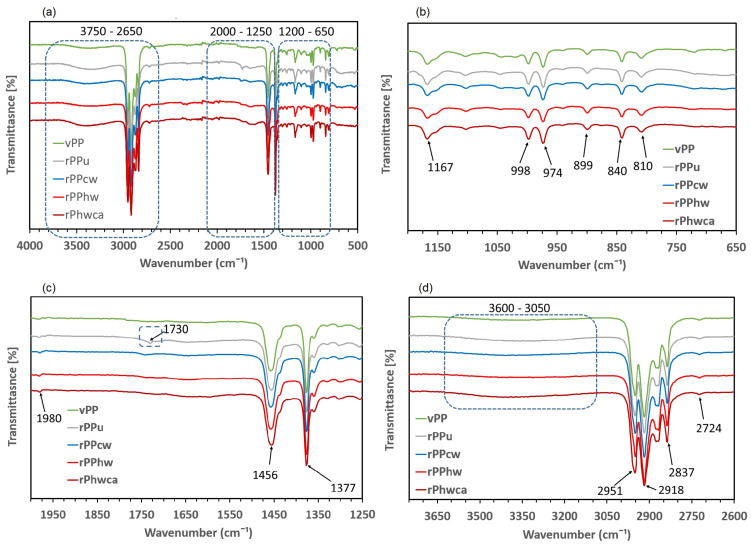
FTIR transmittance spectra for virgin and recycled PP: (**a**) wavelength range: 4000–500 cm^−1^ (**b**) wavelength range: 1200–650 cm^−1^, (**c**) wavelength range: 2000–1250 cm^−1^, (**d**) wavelength range: 3750–2650 cm^−1^.

The FTIR spectra of virgin and recycled PP are shown in Figure 5a, and their detailed presentation in the different wavelength ranges in Figure 5b–d. All spectra are quite similar, displaying the characteristic peaks of PP [44,45] described in more detail in Table 5.

However, some additional intensities of the vibrational bands were detected in some of the samples of the recycled PP under different washing procedures. As shown in Figure 5c, there is a peak at 1730 cm^−1^, assigned to the saturated C = 0 stretch of the carbonyl functional group in rPPu (unwashed sample), indicating polymer degradation. This peak is not detected in vPP; its intensity decreases drastically in rPPcw and disappears in the rPPhw and rPPhwca. These findings are in line with the conclusions of Day et al. [29], who pointed out an acceleration of polymer degradation in the presence of impurities. Moreover, it corroborates the conclusions of Garofalo et al. [7], who reported an attenuation of intensity at 1730 cm^−1^ wavelength for washed mixed polyolefin-based recyclates. As for the broad water vapor band between 3050 cm^−1^ and 3600 cm^−1^, highlighted in Figure 5d with the corresponding stretching vibrations of O=H or N-H [42], it is present in all samples, including vPP, its intensity being higher for PPu and rPPhwca. The slightly higher spectral intensity of the latter samples may be attributed to increased water sorption of the unwashed rPP and, thus, not subjected to drying and thoroughly washed rPPhwca, requiring more exposure to water for effective removal of the cleaning agents.

### 3.4. Mechanical Properties

**Figure 6:**
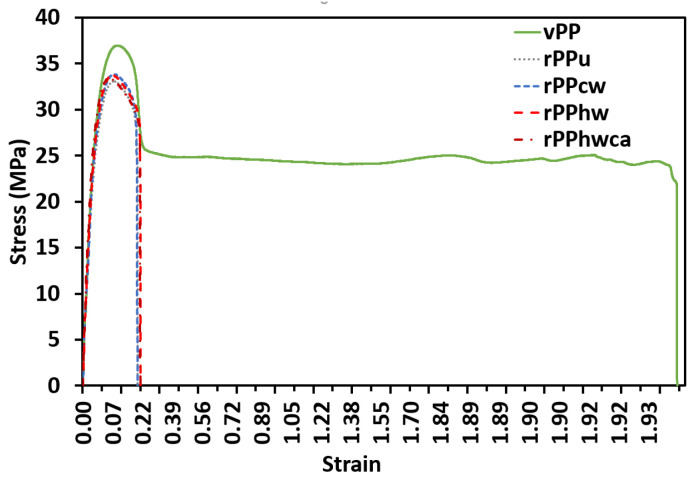
Stress vs. strain response of virgin and recycled PP.

**Figure 7:**
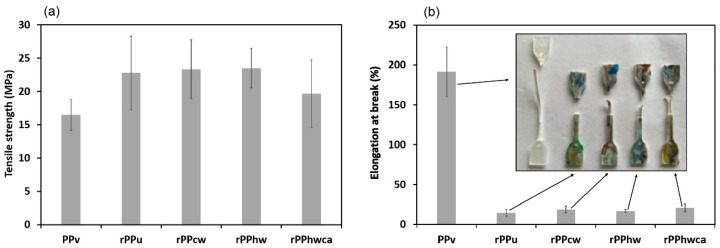
Mechanical properties of virgin and recycled PP: (**a**) tensile strength; (**b**) elongation at break.

The mechanical properties, accessed by tensile testing (E—Young’s modulus; σ_Y_—stress at yield; σ_u_—ultimate tensile strength; ε_b_—elongation at break) are summarized in Table 6. As shown in Figure 6, the recycled PP samples underwent some reduction in the yield stress and a severe decrease in ductility, rupturing almost instantly. The latter may be attributed to the thermo-oxidative degradation of the recycled PP samples, confirmed by the DSC tests, due to scission and disentanglement of the molecular chains in the amorphous phase and reduction of their interconnections with the crystalline regions [46]. A slight increase in the yield stress was observed for rPPcw, rPPhw, and rPPhwca compared with the unwashed rPP, corroborating the thermal, rheological, and FTIR spectroscopy results, which is also in agreement with the findings of Garofalo et al. [7]. The higher stiffness of all recycled PP samples is reflected in the higher ultimate tensile strength (Figure 7a), indicating the significant deterioration of their ductility, as demonstrated in Figure 7b. The elongation values at break for vPP samples comply with the data (>50%) presented in the Moplen HP500N data sheet. As expected, the vPP samples showed the highest elongation (191.41%) until rupture. The worst resistance to rupture among the recycled PP batches was obtained for rPPu, which broke at 14.58% of elongation without any necking. Thus, Figure 7b confirms the influence of contaminants on the reduction of ductility in recycled PP. It was impossible to draw any conclusions about the influence of the degradation and the implemented washing methods on the elastic properties of recycled PP, as the Young modulus varied between the batches from 1379.73 to 1619.88 MPa. The heterogeneity of recycled PP composition may explain this variation and the high dispersity of the results from sample to sample.

## 4. Conclusions

Post-consumer PP waste contamination, heterogeneity, and degradation can complicate reprocessing and the quality of recycled components. Melt flow index measurements unambiguously pointed out the heterogeneity of the recycled PP composition and their constituent materials’ origin (packaging), with MFI varying between 32 to 42 g/10 min, falling within the range of the medium and low viscosity grades. The melt flow index standard deviation was significantly minimized in all washed samples, varying between 1.49 and 4.42 g/10 min, compared with the standard deviation of 13.04 g/10 min of rPPu, indicating an improvement in their processability. Thermal characterization by DSC revealed that the recycled PP, independently of the washing method, required less energy to melt but crystallized under higher temperatures. Both occurrences can be attributed to the degradation processes by accumulating chemical impurities in the form of carbonyl groups resulting from the oxidative processes and reduction of molecular mass due to polymer chain scission. The latter is likely responsible for the lower crystalline content (47–49%) of recycled PP compared with virgin PP (53%). OIT tests demonstrated that for all the recycled PP samples, significantly less time was required to induce oxidation than in the reference vPP sample, indicating some depletion of the initially present antioxidants that protected the polymeric chains from the oxidation processes by preventing their scission. The weakened molecular structure resulted in the drastic reduction of ductility in all samples of recycled PP and, therefore, a significant decrease in the elongation at break (15–21%) compared with virgin PP (191%).

Nevertheless, it should be stressed that a slight ductility improvement was observed in the samples decontaminated by washing. The significance of this observation was confirmed by the FTIR spectroscopy data, where the wave intensity peak at 1730 cm^−1^, assigned to the saturated C = 0 stretch of the carbonyl functional group, was detected in rPPu. The intensity of this peak decreased drastically in rPPcw and disappeared in the rPPhw and rPPhwca, highlighting the efficiency of washing for attenuation of the thermo-oxidative degradation intensity. These results demonstrate that contaminants act as degradation catalysts, and their removal improves the processability and mechanical properties. It can be concluded that the most promising washing procedure for improving the recycled PP quality is cold washing (rPPcw), as it showed very similar results to rPPhw and rPPhwca methods, with the advantages of energy savings and environmental safety.

## Figures and Tables

**Table 1:** Washing and drying conditions of recycled PP.

PP Batch	Washing Temperature (°C)	Drying Temperature (°C)	Cleaning Agents
rPPcw	20	60	none
rPPhw	75	60	none
rPhwca	75	60	Triton X-100 0.3% ^1^ and NaOH 0.5% ^1^

^1^ weight content in water.

**Table 2:** Injection molding processing conditions.

Parameter	Value
Plasticization chamber temperature (°C)	230
Mold temperature (°C)	40
Injection time (s)	3
Injection pressure (bar)	300
Packing time (s)	17
Packing pressure (bar)	240
Plasticization time (s)	180

**Table 3:** Relevant data from DSC thermograms.

Sample	T_m_ (°C)	T_c_ (°C)	ΔH_m_ (J/g)	χ (%)
vPP	165 (±0.31)	113 (±0.38)	109 (±2.41)	53
rPPu	163 (±0.30)	123 (±0.43)	98 (±0.66)	48
rPPcw	162 (±0.21)	122 (±0.16)	99 (±0.54)	48
rPPhw	163 (±0.76)	122 (±0.60)	97 (±2.69)	47
rPhwca	163 (±0.24)	123 (±072)	100 (±1.32)	49

Numbers in parentheses stand for standard deviation.

**Table 4:** Relevant data from DSC thermograms.

Sample	OIT(s)	Standard Deviation
	Mean	±
vPP	274.2	51.98
rPPu	53.0	9.62
rPPcw	41.6	5.54
rPPhw	41.6	1.39
rPhwca	61.4	4.42

**Table 5:** FTIR peak maxima.

Wavenumber (cm^−1^)	Group Vibrations
810	CH2 r, CC s
840	CH2 r
899	CH3 r, CH2 r, CH b
974	CH3 r, CC s
998	CH3 r, CH b, CH2 w
1167	CC s, CH3 r, CH b
1257	CH b, CH2 t, CH3 r
1377	CH3 umbrella bending mode
1456	CH3 b assym., CH2 b
1730	C = O s (carboxylic acid group)
2837	CH2 s sym.
2918	CH2 s assym.
2951	CH3 s assym.
3050–3600	O=H s or N H s

b—bending; r—rocking; t—twisting; s—stretching; w—wagging.

**Table 6:** Mechanical properties of virgin and recycled PP.

Batch	E (MPa)	σ_y_ (MPa)	σ_u_ (MPa)	ε_b_ (%)
vPP	1545.69 (±58.73)	38.33(±1.73)	16.48 (±2.31)	191.41 (±31.27)
rPPu	1459.48 (±170.26)	33.20 (±0.79)	22.78 (±5.52)	14.58 (±4.42)
rPPcw	1379.72 (±59.13)	33.90 (±0.70)	23.32 (±4.45)	18.75 (±4.17)
rPPhw	1619.88 (±160.29)	33.90 (±077)	23.51 (±2.98)	16.97 (±1.79)
rPhwca	1516.02 (±190.92)	33.52 (±0.06)	19.68 (±5.07)	20.76 (±4.92)

Numbers in parentheses stand for standard deviation.

## Data Availability

Not applicable.
